# Supplementary material for: Causal Relationship between Aging and Anorexia Nervosa: A White-Matter-Microstructure-Mediated Mendelian Randomization Analysis
Source: Biomedicines. 2024 Aug 16;12(8):1874. doi: 10.3390/biomedicines12081874 (PMC11351342; doi:10.3390/biomedicines12081874)
Supplement: Supplementary file 1 [file biomedicines-12-01874-s001.zip › Supplementary Figure/Figure S9/Leaveoneout plot of MR analysis for ubm-b-1798 to AN.pdf]

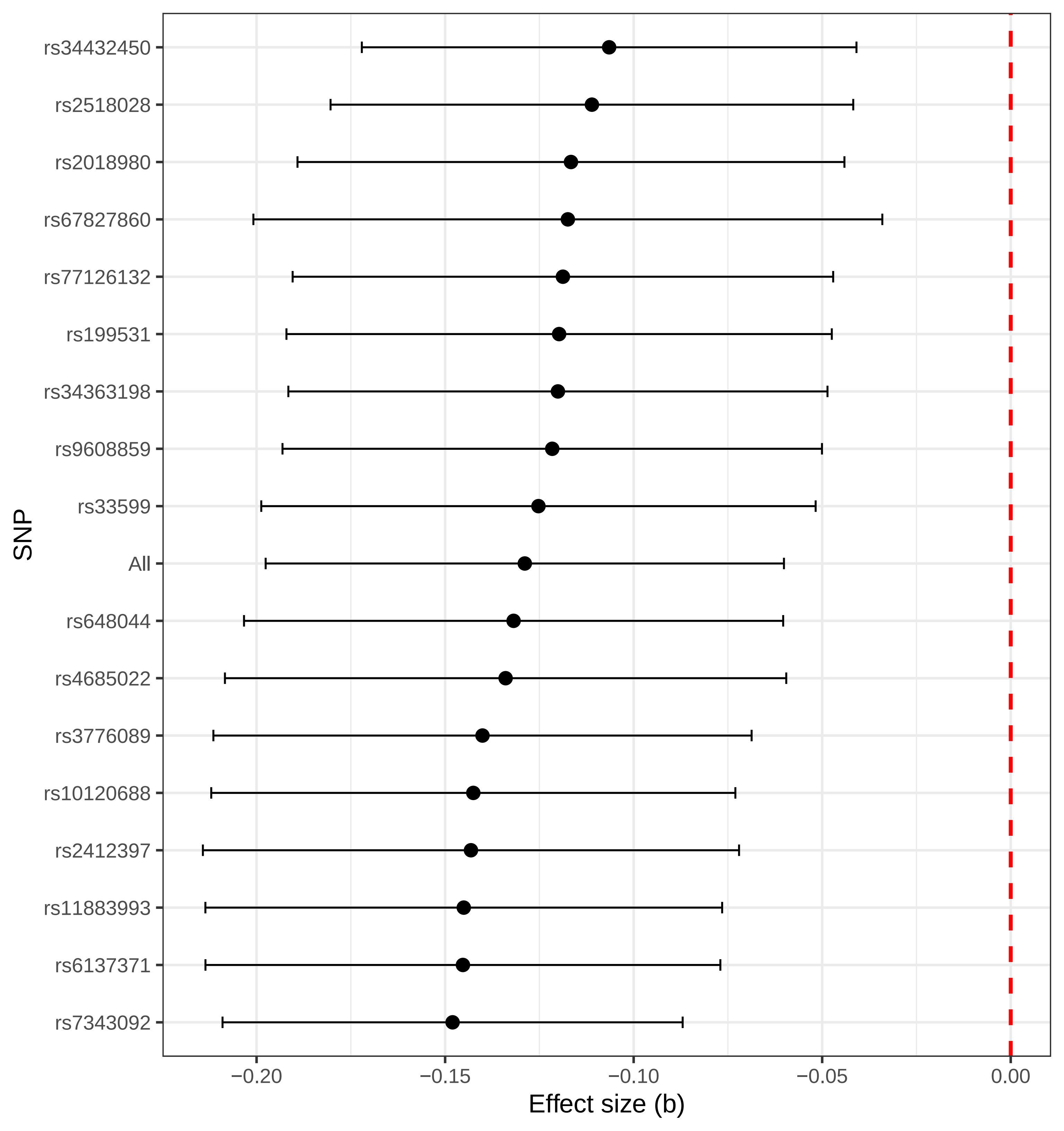
rs34432450
rs2518028
rs2018980
rs67827860
rs77126132
rs199531
rs34363198
rs9608859
rs33599
All
rs648044
rs4685022
rs3776089
rs10120688
rs2412397
rs11883993
rs6137371
rs7343092
SNP
−0.20
−0.15
−0.10
−0.05
0.00
Effect size (b)
